# Feeding Relationship between *Octopus vulgaris* (Cuvier, 1797) Early Life-Cycle Stages and Their Prey in the Western Iberian Upwelling System: Correlation of Reciprocal Lipid and Fatty Acid Contents

**DOI:** 10.3389/fphys.2017.00467

**Published:** 2017-07-19

**Authors:** Sílvia Lourenço, Álvaro Roura, María-José Fernández-Reiriz, Luís Narciso, Ángel F. González

**Affiliations:** ^1^Interdisciplinary Centre of Marine and Environmental Research, Cruise Terminal of the Port of Leixões Porto, Portugal; ^2^Divisão de Serviços de Investigação da Direção Regional das Pescas e Aquacultura da RAM, Centro de Maricultura da Calheta Calheta, Portugal; ^3^Oceanic Observatory of Madeira, Agência Regional para o Desenvolvimento da Investigação Tecnologia e Inovação Funchal, Portugal; ^4^Instituto de Investigaciones Marinas (CSIC) Vigo, Spain; ^5^Department of Ecology, Environment and Evolution, La Trobe University Melbourne, VIC, Australia; ^6^Mare—Marine and Environmental Sciences Centre, Faculdade de Ciências da Universidade de Lisboa Lisbon, Portugal

**Keywords:** *Octopus vulgaris*, paralarvae, fatty acids, lipid content, zooplankton, prey-predator relationship

## Abstract

Under the influence of the Western Iberian upwelling system, the Iberian Atlantic coast holds important hatcheries and recruitment areas for *Octopus vulgaris*. Recently identified as an octopus hatchery, the Ría de Vigo harbors an important mesozooplankton community that supports *O. vulgaris* paralarvae during the first days of their planktonic stage. This study represents a preliminary approach to determine the nutritional link between wild *O. vulgaris* hatchlings, paralarvae and their zooplankton prey in the Ría de Vigo, by analyzing their lipid class content and fatty acid profiles. The results show that octopus hatchlings are richer in structural lipids as phospholipids and cholesterol, while the zooplankton is richer in reserve lipids like triacylglycerol and waxes. Zooplankton samples are also particularly rich in C18:1n9 and 22:6n3 (DHA), that seem to be successfully incorporated by *O. vulgaris* paralarvae thus resulting in a distinct fatty acid profile to that of the hatchlings. On the other hand, content in C20:4n6 (ARA) is maintained high through development, even though the zooplankton is apparently poorer in this essential fatty acid, confirming its importance for the development of *O. vulgaris* paralarvae. The content in monounsaturated fatty acids, particularly C18:1n7, and the DHA: EPA ratio are suggested as trophic markers of the diet of *O. vulgaris* paralarvae.

## Introduction

The common octopus (*Octopus vulgaris* Cuvier, 1797) is the most important commercially harvested octopus worldwide. With global landing estimates of 42,457 ton/year (FAO, 2016), it is consumed in many countries of Asia, Latin-America and Mediterranean. The high market demand and value, along with the biological characteristics like the short life span, high growth rates and high food conversion, makes *O. vulgaris* a desirable species for aquaculture production (Vaz-Pires et al., 2004). However, after decades of research the low paralarvae survival remains an important constraint for industrial farming (Iglesias et al., 2007; Garrido et al., 2016b).

The Iberian Atlantic coast is an important hatchery and recruitment area for *O. vulgaris* (Moreno et al., 2014; Guerra et al., 2015). Here, *O. vulgaris* paralarvae find the optimal environmental conditions to grow favored by the strong summer upwelling (Moreno et al., 2009; Roura et al., 2016). In the first days of life, the paralarvae combine endogenous (yolk) with exogenous feeding, preying mainly upon larval stages of crustaceans of the families Crangonidae, Alpheidae, Brachyura, Paguridae, Thalassinidae, Porcellanidae, Cladocera, Copepoda, and Euphausiidae, but also fish larvae and Cnidaria (Roura et al., 2012; Olmos-Pérez et al., 2017). In fact, during summer, all these potential prey are naturally “enriched” with essential fatty acids (EFA) by the seasonal coastal upwelling where the frequent diatom and dinoflagelate blooms are responsible for the production of polyunsaturated fatty acids (PUFA) as 20:5n3 (eicosapentaenoic acid, EPA) and 22:6n3 (docosahexaenoic acid, DHA). The phytoplankton fatty acid (FA) composition and, particularly, the ratios PUFA (n-3)/(n-6) and EPA/DHA will influence the FA composition of the linked trophic levels like meso- and microzooplankton and planktivorous fishes (Dalsgaard et al., 2003) like *Sardina pilchardus* (Garrido et al., 2008) and to certain extent tissues and eggs of higher trophic levels species as *O. vulgaris* (Lourenço et al., 2014). In fact, the FA composition of muscle and eggs in marine organisms reflects to certain level the biochemical and ecological conditions of ecosystems and can be used to identify food web interactions (Bergé and Barnathan, 2005) being used as qualitative markers, or biomarkers, to trace or confirm predator-prey relationships (Dalsgaard et al., 2003; Budge et al., 2006).

From the metabolic perspective, marine lipids have key roles in the physiology and reproductive processes of heterotrophic organisms. The neutral lipids triacylglycerols and wax esters, are energy reserves that produce free fatty acids through oxidation, which will be incorporated into phospholipids and again in fat reserves (Budge et al., 2006). Phospholipids are the building blocks for the membrane lipid bilayer. The lipids facilitate the absorption of fat-soluble vitamins (e.g., Vitamins A, D, E, and K), and play an important role in the production and regulation of eicosanoids (Bergé and Barnathan, 2005). Cholesterol is the predominant sterol in cephalopod's lipid reserves (Sieiro et al., 2006) and it is precursor of steroid hormones including cortisol, corticosterone, and cortisone. From these, cortisol has an important role in stress responses and is involved in the regulation of the carbohydrates and protein metabolism (Tocher and Glencross, 2015). Despite the low content of lipids in cephalopod body composition (6% dw in muscle, 24% in digestive gland of adults, Sieiro et al., 2006) and 12% dw of the paralarvae (Navarro and Villanueva, 2003), lipids have critical roles in cephalopod metabolism and development (Navarro and Villanueva, 2000; Okumura et al., 2005; Miliou et al., 2006; Seixas et al., 2010; Monroig et al., 2013; Reis et al., 2015). The lipid-rich nervous system of hatchlings represents approximately one quarter of the animal's fresh weight (Navarro et al., 2014) and the long-chain PUFA, namely EPA, DHA, and C20:4n6 (arachidonic acid, ARA) are identified as EFA for cephalopods, particularly in early life-cycle stages (Monroig et al., 2012; Reis et al., 2014). In fact, several studies have suggested that *O. vulgaris* paralarvae require prey of low lipid content, rich in polar lipids, long-chain PUFA, and cholesterol content (Navarro and Villanueva, 2000, 2003; Okumura et al., 2005; Seixas et al., 2008).

Despite the extended knowledge about the environmental physical factors that drive the distribution, abundance, and recruitment success of *O. vulgaris* paralarvae (González et al., 2005; Otero et al., 2008, 2009; Moreno et al., 2009; Roura et al., 2013, 2016), there are few studies regarding the nutritional profile and requirements of wild *O. vulgaris* paralarvae and their natural prey. In recent years, major efforts have been conducted to understand the nutritional needs for paralarvae in captivity (Garrido et al., 2016b) and their fatty acid profile in the wild (Estefanell et al., 2013; Garrido et al., 2016a), however the nutritional link between them and their prey in natural conditions is still largely unknown.

To fulfill this gap, this study aimed to identify the lipid class content of wild *O. vulgaris* hatchlings and paralarvae and that of their potential preys—i.e., the mesozooplankton community—in the Ría de Vigo (NW Spain). The contents in phospholipids, cholesterol, triacylglycerol, free fatty acids, and wax esters were determined in the mezooplankton samples and *O. vulgaris* hatchlings samples. The FA profile was evaluated in the mesozooplankton, hatchlings and paralarvae samples in terms of individual FA, saturated FA (SFA), monounsaturated (MUFA), polyunsaturated (PUFA), n-6 highly unsaturated FA (n-6), and n-3 highly unsaturated FA (n-3). Based in significant dissimilarities analyses, trophic markers were selected and compared between the zooplankton, hatchlings and paralarvae, aiming to understand which FA were incorporated into planktonic *O. vulgaris* paralarvae through their diet.

## Materials and methods

### Zooplankton sampling

**Figure 1:**
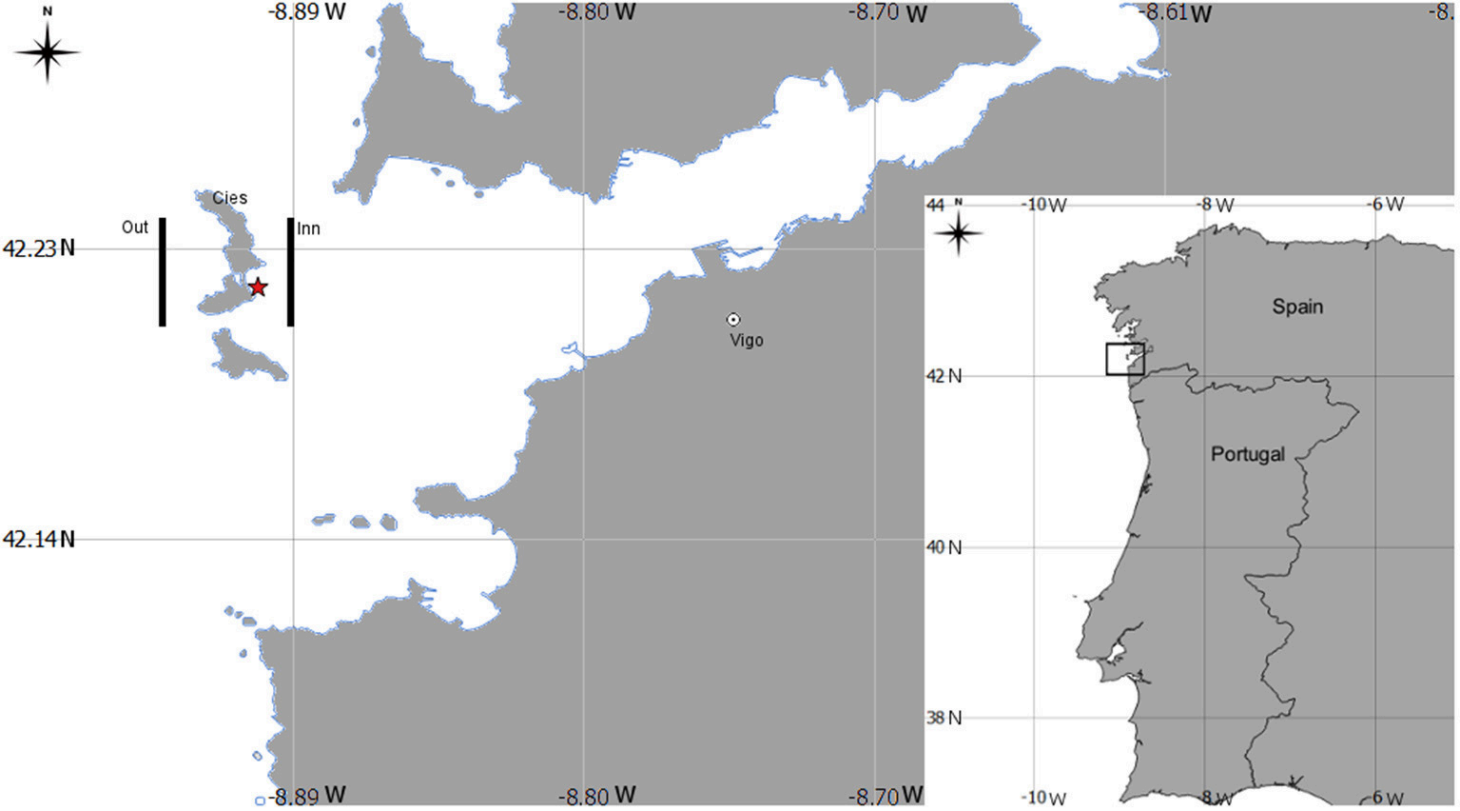
Geographical position of the sampling area in the Ría de Vigo, Spain Northwest Atlantic coast. Legend: lines indicate the sampling transepts at east (inner) and west (out) of Cies Islands. The symbol * indicates the sampling site for the *Octopus vulgaris* paralarvae collected during diving.

A total of 12 mesozooplankton samples were collected at 5 m depth of the Ría de Vigo (NW Spain, Figure 1) in three surveys conducted under the LARECO project (CTM2011-25929) in autumn 2012, September 17th (d1); October 1st (d2); and October 5th (d3) in the outer part of the Ría de Vigo. Samples were collected with a multitrawl (MultiNet®) sampler (0.71 × 0.71 m opening frame, 200 μm mesh), East (inn samples) and West of Cies Islands (outer samples) and visually examined on board, looking for *Octopus vulgaris* paralarvae, which were manually sorted. Six zooplankton samples (*n* = 6) were washed with sea water and filtered with a 1,000 μm sieve and frozen at −80°C, freeze dried during 48 h and stored again at −80°C for further analytical methods (see below). The zooplankton size selection was supported by the evidence that *O. vulgaris* paralarvae feed preferentially upon prey >1 mm (Passarella and Hopkins, 1991; Villanueva, 1994; Villanueva et al., 1996; Iglesias et al., 2006; Roura et al., 2010). The remaining samples were fixed in 70% ethanol and then used to identify the mesozooplankton community cohabiting with the paralarvae. Organisms were identified under a binocular (Nikon SMZ800) or inverted microscope (Nikon Eclipse TS100) to the lowest possible taxonomic level. The community (holoplankton/meroplankton) ratio was determined based in the number of species identified and classified as holoplankton or meroplankton accordingly to Roura et al. (2013).

The *O. vulgaris* paralarvae collected (*n* = 44) were pooled in a single sample and stored at −20°C in a Methanol: Dichloromethane (2:1) solution to avoid long-term degradation of lipids.

To determine the basal biochemical profile of *O. vulgaris* paralarvae before external feeding, newly hatched paralarvae (hereafter called hatchlings) were obtained from ripe eggs from a single female batch collected by scuba diving in October 9th 2012 off the Ría de Vigo (site coordinates: 42°14′N, 8° 54′W, Figure 1). The hatchlings were pooled and analyzed in duplicate.

### Biochemical methods

Lipids were first extracted from each zooplankton samples and from the single hatchlings with chloroform: methanol (1:2) and after centrifugation, the precipitate was re-extracted with chloroform: methanol (2:1). Both supernatants were subsequently washed with chloroform: methanol: water (8:4:3) as described by Fernández-Reiriz et al. (1989). Total lipids were quantified following the method described by Marsh and Weinstein (1966) with a tripalmitine standard (Sigma Aldrich Inc., Buchs, Switzerland). Wax esters (WAXES), triglycerides (TAG), free fatty acids (FFA), cholesterol (CHL), and phospholipids (PL) content were determined by thin-layer chromatography (TLC)/densitometry. Silica gel 60 W plates (Merck 16486), with a size of 20 × 20 cm and a layer thickness of 0.25 mm, were used. Samples were applied by automatic TLC sampler (Camag 27220). The chromatographic staining was conducted accordingly to Freeman and West (1966). The plates were stained with a 10% CuSO4 solution in 0.85% H_3_PO_4_ by heating to 180°C (Bitman and Wood, 1982). Standards employed for the quantitative analysis of the WAXES, TAG, FFA, and CHOL were oleyl oleate, triolein, oleic acid, and cod liver oil (CHOL, Sigma), respectively. A standard obtained from *Mytilus galloprovincialis* was used for PL. The plates were scanned with a Shimadzu CS9000 densitometer, using a monochromatic 370 nm beam of 0.4 × 0.4 mm working in the zigzag mode, reading the whole spot, and with automatic autozero for baseline correction. All solvents, reagents and fatty acid standards used in this work were of analytic grade (Merck, Darmstadt, and Sigma). FA content of total lipids fraction of zooplankton, *O. vulgaris* hatchlings and paralarvae was determined converting total lipids into FA methyl esters (FAME), accordingly to the method described by Lepage and Roy (1984). Fatty acids methyl esters (FAME) were analyzed by gas chromatography. Peaks corresponding to FAME were identified by comparison of their retention times with standard mixtures and the concentration of each fatty acid or fatty acid group was expressed as % FAME.

### Statistical analysis

Zooplankton samples were identified according to the correspondent transect (out or inn) and sampling day (d1, d2, and d3) resulting in the following sampling code: Out_d1, out_d2, out_d3, inn_d1, inn_d2, and inn_d3. The zooplankton sample composition, lipid classes and FA content were analyzed using metric multidimensional techniques aiming to identify dissimilarities between groups. Prior to analysis, zooplankton abundance data was transformed log (x+1) and screened to select the taxa that appeared at least in 10% of the samples. Zooplankton dissimilarity matrix was calculated using the Bray-Curtis dissimilarity index and analyzed with principal coordinate analysis (PCO). The species with highest correlation with the first and second coordinate axes were identified as the potential prey group for the lipid analysis (species highlighted in Table 1). The lipid class content and FA with mean concentration higher than 1% FAME were normalized, the similarity matrix was determined using Euclidean distance and analyzed with principal component analysis (PCA) (Zuur et al., 2007). The dimension (axes) eigenvalues and FA scores in each dimension obtained were used to select the FA that explained most of the variance (FA in bold in Table 2). The zooplankton species, lipid class and FA groups identified were tested for differences related with sampling area and species composition by non-parametric permutational ANOVA (PERMANOVA) considering type I errors. A constrained canonical analysis (CCA) was applied to the set of zooplankton prey using FA as explanatory variables to identify significant correlations between these FA and the zooplankton species.

**Table 1:** Mesozooplankton community abundance (n/1,000 m^3^) and % (in parenthesis).

	**species code**	**out_d1**	**out_d2**	**out_d3**	**inn_d1**	**inn_d2**	**inn_d3**
**HOLOPLANKTON**							
*Acartia clausi*	cop_ac	186 (6.93)	90 (6.52)	288 (10.87)	162 (5.25)	549 (24.05)	468 (18.27)
*** Calanoides carinatus***	**cop_cca**	**72 (2.68)**	**24 (1.74)**	**6 (0.23)**	**24 (0.78)**	**18 (0.79)**	**3 (0.12)**
*** Calanus helgolandicus***	**cop_ch**	**6 (0.22)**	**12 (0.87)**	**12 (0.45)**	**12 (0.39)**	**3 (0.13)**	
*Centropages chierchiae*	cop_cch	3 (0.11)				3 (0.13)	
*** Clausocalanus*** **spp**.	**cop_cla**	**48 (1.80)**	**97 (0.65)**	**3 (0.11)**	**21 (0.68)**	**9 (0.39)**	
*** Corycaeu*****s spp**.	**cop_cor**		**18 (1.30)**	**3 (0.11)**		**24 (1.05)**	**36 (1.41)**
*Paracalanus parvus*	cop_pp	624 (23.30)	327 (23.70)	390 (14.72)	183 (5.93)	225 (9.86)	372 (14.52)
*** Paraeuchaeta hebes***	**cop_peh**	**54 (2.01)**	**3 (0.22)**				
*Paraeuchaeta* sp.	cop_pe	3 (0.11)		6 (0.23)			3 (0.12)
*** Ctenocalanus vanus***	**cop_cv**	**30 (1.12)**	**6 (0.43)**	**24 (0.91)**		**24 (1.05)**	
*Pseudocalanus elongatus*	cop_pce	6 (0.22)	6 (0.43)	12 (0.45)	24 (0.78)	30 (1.31)	3 (0.12)
*Subeucalanus crassus*	cop_sec	3 (0.11)		3 (0.11)		9 (0.39)	3 (0.12)
*** Temora longicornis***	**cop_tl**	**21 (0.78)**	**3 (0.22)**	**93 (3.51)**	**57 (1.85)**	**9 (0.39)**	**3 (0.12)**
*Candacia armata*							3 (0.12)
*Oithona plumifera*		3 (0.11)	3 (0.22)	12 (0.45)	12 (0.39)	12 (0.52)	6 (0.23)
*Oncaea media*		21 (0.78)		291 (10.99)	99 (3.21)	6 (0.267)	
Harpacticoida					3 (0.10)	6 (0.26)	3 (0.12)
Copepodid stages	cop	72 (2.68)	42 (3.04)		78 (2.53)	45 (1.97)	6 (0.23)
*Evadne nordmanni*		306 (11.41)	63 (4.57)	9 (0.34)	831 (26.92)	156 (6.83)	54 (2.11)
*Podon intermedius*			15 (1.09)		21 (0.68)	42 (1.84)	33 (1.29)
*Nyctiphanes couchii* calyptopa		51 (1.90)	102 (7.39)	600 (22.65)	144 (4.66)	48 (2.10)	24 (0.94)
*Nyctiphanes couchii* furcilia	nyc_cou	300 (11.18)	129 (9.35)	99 (3.74)	69 (2.24)	69 (3.02)	222 (8.67)
Mysidacea	mys	3 (0.11)		3 (0.11)			
** Cnidaria**	**cnid**			**27 (1.02)**	**15 (0.49)**		**81 (3.16)**
Chaetognatha	chaet	108 (4.03)	21 (1.52)	72 (2.72)	66 (2.14)	84 (3.68)	90 (3.51)
** Syphonophora**	**syph**	**33 (1.23)**		**30 (1.13)**	**96 (3.11)**		
Tunicata	salp	81 (2.93)	12 (0.86)	30 (1.14)	117 (3.65)	63 (2.68)	156 (5.74)
Platyhelminthes							12 (0.47)
**MEROPLANKTON**							
Amphioxus		3 (0.11)					
Gammaridea					6 (0.19)	3 (0.13)	3 (0.12)
Cirripida cipris	cirripid	57 (2.12)	3 (0.22)		27 (0.87)	15 (0.66)	24 (0.94)
Polichaeta larvae	polich		9 (0.65)	12 (0.45)	6 (0.19)	12 (0.53)	3 (0.12)
Bivalvia larvae		429 (16.00)	381 (27.61)	312 (11.78)	699 (22.64)	582 (25.49)	642 (25.06)
Gastropoda		57 (2.13)	69 (5.00)	117 (4.41)	84 (2.72)	138 (6.04)	243 (9.48)
Ophiuridea larvae		45 (1.68)	3 (0.22)	3 (0.11)	159 (5.15)	6 (0.26)	
Equinoidea larvae					9 (0.29)		
Cirripida nauplius		18 (0.67)	9 (0.65)	171 (6.46)	51 (1.65)	27 (1.18)	21 (0.82)
Brachyura zoeae	brach_zoea	30 (1.13)	6 (0.43)	15 (0.57)	6 (0.19)	24 (1.05)	18 (0.70)
Crangonidae zoeae	crang_zoea					12 (0.53)	
*** Paguridae*** **zoeae**	**pag_zoea**				**3 (0.10)**		**6 (0.23)**
*** Palaemonidae*** **zoeae**	**palam_zoea**			**3 (0.11)**		**6 (0.26)**	**3 (0.12)**
Bryozan larvae		6 (0.22)	12 (0.87)			15 (0.66)	9 (0.35)
*** Pisidia longicornis*** **zoeae**	**p_long_zoea**					**3 (0.13)**	**3 (0.12)**
*Porcellana platycheles* zoeae	p_platy_zoea	3 (0.11)	3 (0.22)				6 (0.23)
*Processidae* zoeae	process_zoea				3 (0.10)	6 (0.26)	
Fish eggs				3 (0.11)			
Fish larvae						3 (0.13)	
Holoplankton/Meroplankton		1.91	1.77	1.83	2.56	2.11	3.14

*Out_d1, out_d2, out_d3, inn_d1, inn_d2, and inn_d3 represent the zooplankton samples. Zooplankton species with individuals bigger than 1 mm were selected as prey for Octopus vulgaris paralarvae and analyzed for their nutritional profile (identified with a species code). The species in bold were selected for the constrained canonical analysis (CCA)*.

**Table 2:** Fatty acid concentration (mean ± SD, % FA) of zooplankton community and *Octopus vulgaris* hatchlings in the Ría de Vigo.

	**Mesozooplankton**	***O. vulgaris***	
		**Hatchlings**	**Paralarvae**
**Saturated fatty acids (SFA)**			
C14:0	5.46 ± 0.84^a^	2.47 ± 0.17^b^	2.33
C15:0	0.63 ± 0.10^a^	0.33 ± 0.03^b^	0.65
C16:0	18.88 ± 1.21^a^	19.35 ± 1.46^b^	21.97
C17:0	1.35 ± 1.18^a^	1.42 ± 0.11^b^	1.49
C18:0	4.57 ± 0.41^a^	9.96 ± 0.75^b^	10.58
C24:0	0.77 ± 0.11^a^	0.65 ± 0.06^a^	
ΣSFA	31.69 ± 6.97^a^	34.19 ± 2.59^a^	37.02
**Monounsaturated fatty acids (MUFA)**			
C15:1		0.37 ± 0.04^b^	0.46
C16:1n7	7.18 ± 1.10^a^	0.54 ± 0.05^b^	1.31
C17:1	1.21 ± 0.20^a^	3.29 ± 0.26^b^	3.03
C18:1n7	3.15 ± 0.50^a^	1.67 ± 0.19^b^	1.94
C18:1n9	4.61 ± 0.51^a^	2.82 ± 0.25^b^	7.65
C20:1n9	0.73 ± 0.41^a^	4.09 ± 0.29^b^	4.61
C22:1n9	0.62 ± 0.66^a^	0.90 ± 0.14^a^	
C24:1n9	0.65 ± 0.09^a^	0.52 ± 0.03^a^	0.63
Σ MUFA	19.40 ± 2.41^a^	15.66 ± 1.38^b^	21.10
**Poli-unsaturated fatty acids (PUFA)**			
C18:2n6	1.78 ± 0.04^a^	0.37 ± 0.02^a^	0.67
C18:4n3	2.61 ± 0.28^a^	0.39 ± 0.03^b^	
C18:3n3	1.41 ± 0.12		
C20:2n6		0.76 ± 0.07	
C20:4n6^1^(ARA)	1.79 ± 0.19^a^	5.32 ± 2.40^b^	5.06
C20:4n3	0.81 ± 0.12		
C20:5n3 (EPA)	22.23 ± 1.51^a^	18.43 ± 1.44^b^	15.40
C22:5n3	0.90 ± 0.05^a^	1.63 ± 0.13^b^	1.78
C22:6n3 (DHA)	17.34 ± 2.69^a^	23.25 ± 1.78^b^	18.97
Σ PUFA	50.17 ± 8.17^a^	50.15 ± 5.86^a^	41.88
Σn-6	3.57 ± 0.16	6.37 ± 1.63	5.73
n-3/n-6	12.74 ± 0.16	7.27 ± 2.33	3.62
DHA/EPA	0.79 ± 0.16	1.26 ± 0.00	1.23
EPA/ARA	12.47 ± 0.76	3.85 ± 1.56	3.04
DHA/ARA	9.88 ± 2.36	4.87 ± 2.00	3.75

1 *The FA C20:4n6 and FA C20:3n3 have the same retention time, and the concentration of FA C20:4n6 is dominant in marine products, the concentration presented here is representative of C20:4n6*.

*Different superscripts indicate significant statistical differences (p < 0.05) between mesozooplankton, O. vulgaris hatchlings and paralarvae*.

Following, the lipid class and FA content were compared between zooplankton, *O. vulgaris* hatchlings and paralarvae applying PCA to determine which lipid classes and FA could differentiate between the groups. The groups identified were tested with PERMANOVA for sample type and sample site to test the significance among groups. CCA was applied to the set of most influential FA, using selected trophic markers as explanatory variables to identify significant differences between the FA profile of prey, hatchlings and paralarvae and the zooplankton species selected. The trophic markers selected were ΣSFA (SFA), ΣMUFA (MUFA), ΣPUFA (PUFA), Σn-6HUFA (n-6), Σn-3HUFA (n-3), SFA/PUFA; n-3/n-6; DHA/ARA; DHA/EPA). The metric multidimensional analysis was conducted applying the “envfit” function of VEGAN package in R (Oksanen et al., 2013).

## Results

**Figure 2:**
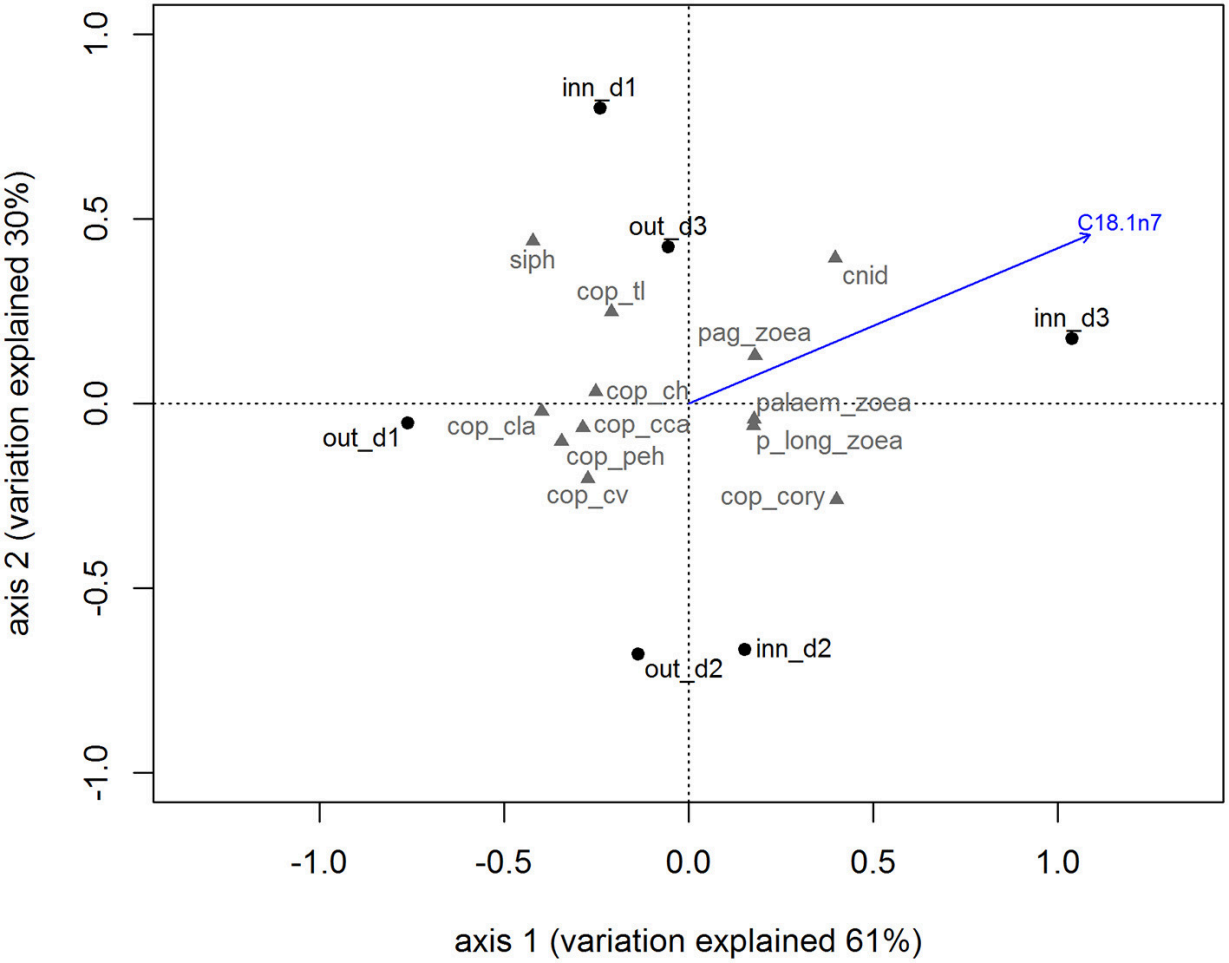
Biplot for principal component analysis of zooplankton community accordingly with sampling site. The blue vector represents the most correlated variable obtained by canonical constrained analysis. Out_d1, out_d2, out_d3, inn_d1, inn_d2, and inn_d3 represent the zooplankton samples scores and the gray codes represent the zooplankton species scores (see Table 1 for species names).

The species composition of the zooplankton in the Ría de Vigo (Table 1) showed a dominance of holoplankton both in the inner zone (67.65%) and the outer zone (83.61%) with the copepods *Paracalanus parvus, Acartia clausii*, and the euphausid *Nyctiphanes couchii* being the most frequent species. The meroplankton species contributed with 32.35% in the inner zone and 16.39% in the outer zone, with the most frequent larvae being bivalves and gastropod larvae and cirripeds, mainly in the inner zone stations. The holo/meroplankton ratio of the zooplanktonic community ranged between 1.77 in the outer zone and 3.14 in the inner zone indicating that the two sampling groups belonged to the same coastal community. The inner and outer zone zooplankton communities presented similar total lipids and lipid class content, only differing in the concentration of a single FA. The FA C18:1n7 is particularly high in the zooplankton community of the inner zone (blue arrow in the Figure 2). The correlation results showed that C18:1n7 was highly correlated with zoaea of different crustaceans and cnidarians.

**Figure 3:**
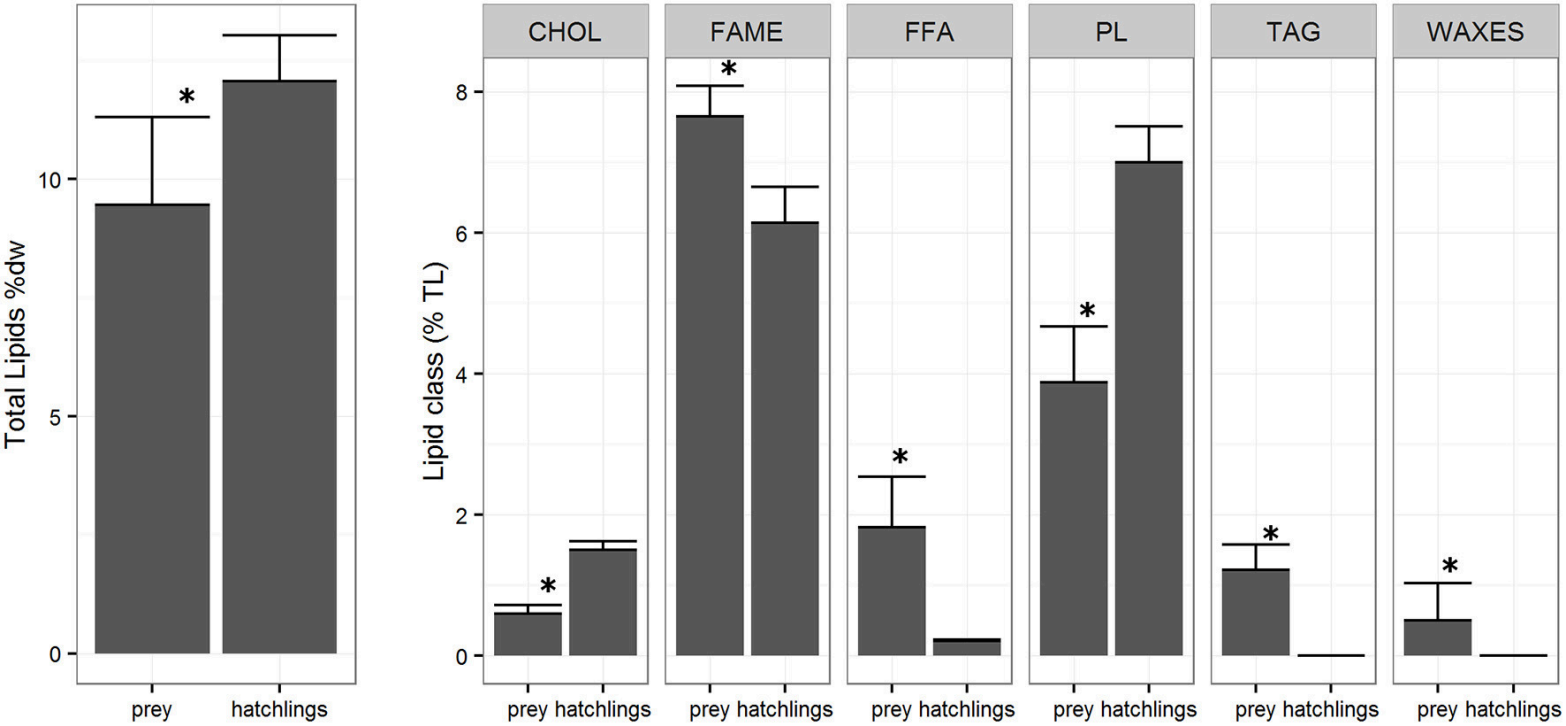
Zooplankton and *Octopus vulgaris* hatchlings total lipids (TL %) (± SD) and lipid classes (% TL) (± SD). ^*^Indicates a significance level of *p* < 0.05 between groups.

The lipid class composition of *Octopus vulgaris* hatchlings was significantly different of that of the zooplankton community (Figure 3). The *O. vulgaris* hatchlings were richer in PL, followed by CHOL and low content in FFA and no TAG and WAXES were detected. In general, hatchlings are richer in FA than zooplankton, in detail, the FA profile of both zooplankton and hatchlings (Table 2) showed that, while the two groups had similar content of ΣSFA and ΣPUFA, the zooplankton had higher content of ΣMUFA, particularly in C16:1n7, C18:1n7, and C18:1n9. Despite the similarity in the ΣPUFA, zooplankton samples were richer in EPA, while hatchlings and paralarvae had higher content in ARA and DHA.

**Figure 4:**
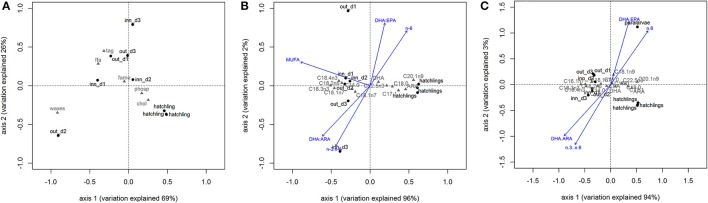
Biplots of principal component analysis of correlation between zooplankton and *Octopus vulgaris* hatchlings based in lipid classes content **(A)** and fatty acid profile **(B)**. Biplot C represents the principal component model comparing the fatty acid profile of zooplankton samples with *Octopus vulgaris* hatchlings and paralarvae. The vectors represent the most correlated trophic markers from canonical constrained analysis. Legend: out_d1, out_d2, out_d3, inn_d1, inn_d2, and inn_d3 represent site score for zooplankton samples, the gray codes represent lipid classes scores **(A)** and fatty acid scores **(B,C)**.

PCA showed that the lipid class content allowed to separate zooplankton samples from *O. vulgaris* hatchlings, explaining 95% of the model variation (Figure 4A) supported by PERMANOVA, *F* = 6.67, *p*-value = 0.025, 999 perm). The *O. vulgaris* hatchlings were correlated with higher content of CHOL, while the zooplankton samples were correlated with higher content in TAG, FFA, and WAXES (particularly the sample out_d2). Comparing the FA profile of *O. vulgaris* hatchlings with the zooplankton samples, most FA showed different concentrations with exception of C24:0, C22:1n9, C24:1n9, and C18:2n6. Some FA were only identified in *O. vulgaris* hatchlings as the C18:1n9 and C20:2n6, while others were only identified within the zooplankton samples, like C18:3n3 and C20:4n3. The overall FA profile is significantly different when comparing the zooplankton and the *O. vulgaris* hatchlings (PERMANOVA, *F* = 139.29, *p*-value = 0.01, 999 perm). The biplot in Figure 4B shows that the first axis explained about 96% of the variation observed and the zooplankton samples were correlated with higher content of short-chain C14:0, C16:1n7, and the family of C18:0. However C18:0 was positively correlated with *O. vulgaris* hatchlings, as well as the long-chain FA C20:1n9, C22:5n3, and ARA, and the MUFA C17:1.

By comparing the zooplankton samples with *O. vulgaris* hatchlings, some differences arose. The trophic markers selected to compare zooplankton with *O. vulgaris* hatchlings showed significant differences between the two groups (blue arrows in Figure 4B), particularly MUFA, n-3/n-6, DHA/ EPA, and DHA/ARA. For instance, n-3/n-6 is two times higher in the zooplankton prey (12.74 ± 0.99) than in *O. vulgaris* hatchlings (7.27 ± 2.33), which influences in the same degree the DHA/ ARA (zooplankton 9.88 ± 2.36; hatchlings 4.87 ± 2.00).

The FA profile identified in the sample of 40 paralarvae collected in the wild showed that some of the minority FA (<1% FAME) identified in hatchlings were not identified in this older stage (Table 2). It is noteworthy that C16:1n7 and C18:1n9 contents were particularly high in the paralarvae in comparison with that of the hatchlings. The ARA content was identical in both, hatchlings and paralarvae, and higher than the zooplankton samples. The DHA content of paralarvae was identical to the zooplankton and lower to that of the hatchlings. EPA content in wild paralarvae was particularly low in comparison with the other groups. Overall, planktonic *O. vulgaris* showed higher concentrations of ΣMUFAs and lower concentrations of ΣPUFAs when compared with hatchlings. The PCA reflected those differences separating the planktonic paralarvae from hatchlings and zooplankton samples, mainly based in the differences in the content of C18:1n9 (Figure 4C). PERMANOVA results showed that the FA profile is different between these three groups in terms of both FA identified and FA content (*F* = 85.08, *p*-value = 0.004, 999 perm). The trophic markers (blue arrows in the Figure 4C biplot) were highly correlated with axis 1 (95% explained variation), indicating that differences found in these trophic markers ratios were more significant between *O. vulgaris* samples and zooplankton samples than between hatchlings and paralarvae.

## Discussion

This study represents the first attempt to analyse the FA contents of *O. vulgaris* paralarvae and that of the zooplankton community where they fed on during the first days of their planktonic life. Moreover, a detailed description of the lipid class composition of wild *O. vulgaris* hatchlings and zooplankton was carried out to understand how they differ. Being aware of the seasonal, regional, and sampling limitations, this study still represents an important snapshot on the nutritional support provided by the zooplanktonic community to the *O. vulgaris* paralarvae. *Octopus vulgaris* paralarvae are lecithotrophic and in the first days of life, their survival depends of the embryonic yolk which nutritional composition is directly influenced by female's diet (Quintana et al., 2015). After some hours or a few days in the water column the paralarvae start to feed, and with 7 days-old (Garrido et al., 2016a) they are able to feed in a large variety of prey from decapod zoaea, krill, fish larvae, cladocerans, copepods, siphonophores, and jellyfish (Roura et al., 2012; Olmos-Pérez et al., 2017). Here, we observed that the FA profiles of wild *O. vulgaris* hatchlings and paralarvae are different from those of the zooplankton. Given that the zooplankton samples analyzed were constituted by numerous phyla, with different FA and lipid class compositions (Dalsgaard et al., 2003), this difference may be the result of the trophic selection displayed by *O. vulgaris* paralarvae (Roura et al., 2016).

The zooplanktonic samples analyzed during this study included a heterogeneous assemblage of organisms dominated by two copepods *Paracalanus parvus* and *Acartia clausi*, the euphausiid *Nyctiphanes couchii*, chaetognaths and small Tunicata. This assemblage was particularly rich in FFA, TAG, and WAXES. The zooplankton accumulates TAG and WAXES, important energy reserves produced by the microalgae during the frequent upwelling events (Lee et al., 2006). The higher content in TAG in these samples is probably related with the presence of meroplankton species in some samples, particularly cirripeds and brachyuran larvae that are known to storage TAG in large lipid globules (Lee et al., 2006), in opposition to the copepod dominated samples richer in WAXES (Lee et al., 1970, 1971).

This zooplankton community was rich in SFA and PUFA because of the dominance of calanoid copepod species. The higher availability of bacteria, detritus, and green algae during autumn may account for the increase of the content in SFA (~30%) and PUFA (~49%) (Falk-Petersen et al., 2002; Gonçalves et al., 2012). Moreover, MUFA, particularly C18:1n7 had an important role in the nutritional characterization of the zooplankton (see Figures 2, 4B). Despite the relatively low content in comparison with other FA like C18:1n9, increasing concentrations of C18:1n7 might be related with higher abundance of the meroplankton fraction in the zooplankton samples, which is characteristic of coastal communities (Roura et al., 2013). We suggest that this FA can be used as a trophic marker evaluating the contribution of holoplankton and meroplankton to the *O. vulgaris* paralarvae diet.

Several authors have previously shown that newly hatched paralarvae have low lipid content with relatively high PL and CHOL and very low TAG contents (Navarro and Villanueva, 2000; Reis et al., 2015). In comparison with the zooplankton samples, the hatchlings sample presented higher content of PL and CHOL, and lower content in FFA. The CHOL and PL, important components of cell membranes, have origin in the maternal reserves (Quintana et al., 2015) explaining their relative high content in the hatchlings. On the other hand, we couldn't detect WAX and TAG in hatchling samples, suggesting a very low content as observed in the work of Navarro and Villanueva (2000). These results show that besides the total lipid contents, is the proportion and content of some lipids classes that have high relevance for the paralarvae (Navarro et al., 2014; Reis et al., 2015). In this transitional phase, the digestive gland is still developing (Moguel et al., 2010) and is not able to store and digest the neutral lipids as TAG and WAX until 12 days after hatching (Martínez et al., 2011), explaining why, despite being highly energetic nutrients, these lipid classes appear in a very low concentration in the paralarvae. In fact, previous studies on paralarvae nutritional requirements that used *Artemia* as live feed, seem to have produced paralarvae with important shifts from the natural nutritional profile of the paralarvae (including high TAG content), resulting in high paralarvae mortality probably due to the poor essential lipid composition of the *Artemia* (Navarro et al., 2014).

Capturing *O. vulgaris* paralarvae in zooplankton samples is challenging, as it occurs with many other cephalopod paralarvae with pelagic stages (Moreno et al., 2009; Roura et al., 2016). *Octopus vulgaris* paralarvae are among the less abundant meroplanktonic organisms in the zooplanktonic community (Roura et al., 2013; Zaragoza et al., 2015) and it is very difficult to collect high numbers of individuals to conduct biochemical analyses. To overcome this problem, the approach adopted in the present study was to pool all the paralarvae collected in a unique sample, losing individual information. Alternatively, Garrido et al. (2016b) using the same collection method (the multinet sampler) analyzed 10 *O. vulgaris* paralarvae individually, resulting in high variability between individuals. Both approaches are valid, however, some differences arise, particularly in the content of C18:1n9, and DHA with obvious reflection in the content in Σ MUFA and Σ PUFA. To decrease the uncertainty associated to the FA profiles obtained from *O. vulgaris* paralarvae from nature, the sampling approach could be improved by conducting triplicate field samples of pooled paralarvae collected under the same environmental conditions. However, this approach would only be viable by means of increasing the chance of collecting paralarvae. This could be achieve by filtering more water using bongo nets (González et al., 2005; Roura et al., 2016) or by using light traps, which probed to be quite effective in capturing octopod paralarvae off the NW coast of Australia (Jackson et al., 2008).

DHA and C18:1n9 and are essential for *O. vulgaris* paralarvae (Monroig et al., 2013; Reis et al., 2015) and the difference found between this and the study conducted by Garrido et al. (2016b) might be associated with the high variability in mesozooplankton community composition (Roura et al., 2013), together with the variety of prey hunt by the paralarvae (Roura et al., 2012; Olmos-Pérez et al., 2017). High C18:1n9 is common in neutral lipids (e.g. TAG, Viciano et al., 2011) accumulated by decapod zoaea (see Figure 3; Letessier et al., 2012), one of the preferential prey of *O. vulgaris*, while DHA is associated with dinoflagellate blooms (Dalsgaard et al., 2003) common during autumn (Crespo et al., 2008) and probably dominated in the plankton community during our sampling season.

In marine larvae, SFA and MUFA are the main substrates to incorporate neutral lipids as TAG to satisfy energy demands, while long chain PUFA are preferentially esterified in structural lipids as the phospholipids in cell membranes (Reis et al., 2015). In this study, the paralarvae had higher content of C16:0, C18:0, C16:1n7, and C18:1n9 than hatchlings. This accumulation in SFA and MUFA was probably related with the diet rich in decapod zoaea and other omnivorous and carnivorous holo and meroplankton rich in TAG, consequently in TAG and MUFA (Dalsgaard et al., 2003; Lee et al., 2006). Moreover, ARA content of the paralarvae was similar to that observed in the hatchlings and significantly higher to the prey. The high ARA content was already observed in the mature ovary of females (Rosa et al., 2004; Lourenço et al., 2014; Estefanell et al., 2015) and in hatchlings collected off the Gran Canaria Island (Estefanell et al., 2013). Reis et al. (2015) proved that ARA is efficiently incorporated by the paralarvae. In fact, exists a competition mechanism of incorporation of ARA and EPA that are esterified by the same enzymes, and it is this mechanism that is responsible of the high variability in the EPA/ARA obtained for paralarvae in different studies ranging between 0.95 (for paralarave fed with *Grapsus adcensionis* in Reis et al., 2015), 2.7 in Garrido et al. (2016b), and 3.04 in the present study.

Trophic markers are used to follow the interactions between prey and predators in the marine trophic marine web. In this study, CCA results showed that ratio of essential DHA/ ARA, DHA/ EPA, EPA/ ARA, n-3/ n-6, SFA/ PUFA ratios and the content of ΣMUFA and Σn-6 allowed the discrimination between preys and predators (Budge et al., 2006). In this context, it would be expected to find similar trophic ratios between prey (zooplankton) and predators (paralarvae). In fact, only the paralarvae content in ΣMUFA, Σn-6 and the DHA: EPA ratio seemed to follow the prey composition. As occurs with their prey, *O. vulgaris* paralarvae seem to have a lower content in DHA and higher content in ΣMUFA, presenting the same tendency presented by feeding experiments where *O. vulgaris* hatchlings were fed with known prey (Iglesias et al., 2013; Reis et al., 2014). It is noteworthy, that the MUFA C18:1n7 and C18:1n9 showed an increase in relation to hatchlings following the pattern of their prey.

Even though the low number of samples analyzed, we believe that the lipidic profile and trophic ratios determined for *O. vulgaris* hatchlings, paralarvae and their potential prey, allowed a first approach to understand the impact of the available prey pool in the nutritional profile (in terms of lipids) of *O. vulgaris* paralarvae. The impact of feeding in the FA content, particularly C18:1n7, C18:1n9, and DHA is notable, showing that ΣMUFA, DHA/ EPA, and C18:1n7 can potentially be used as trophic markers of the diet of *O. vulgaris* paralarvae in the wild. Further biochemical and physiological studies targeting the neutral and polar lipid reserves of wild paralarvae and their prey will certainly untangle the nutritional deficiencies obtained under culture conditions for *O. vulgaris* paralarvae.

## Author contributions

SL: Contibuted with the conception, sampling design of the work, and with acquisition, analysis, and interpretation of data, manuscript drafting and preparation for submission. ÁR: Contributed to the work with acquisition, analysis, and interpretation of the data, with critical revision of the manuscript, and final approval for the paper submission. MF: Contributed to the present work with the samples biochemical analysis and interpretation, and for the manuscript critical revision in all aspects considering the lipidic analysis. LN: Contributed for the work conception, critical revision and final approval of the version to be published. ÁG: Contributed for the work conception, critical revision, and final approval of the version to be published.

### Conflict of interest statement

The authors declare that the research was conducted in the absence of any commercial or financial relationships that could be construed as a potential conflict of interest.

## References

[B1] Bergé J.-P. Barnathan G. (2005). Fatty acids from lipids of marine organisms: molecular biodiversity, roles as biomarkers, biologically active compounds, and economical aspects, adv. Biochem. Eng. Biotechnol. 96, 49–125. 10.1007/b135782 16566089

[B2] Bitman J. Wood D. L. (1982). An improved copper reagent for quantitative densitometric thin-layer chromatography of lipids. J. Liq. Chromatogr. 5, 1155–1162. 10.1080/01483918208067575

[B3] Budge S. M. Iverson S. J. Koopman H. N. (2006). Studying trophic ecology in marine ecosystems using fatty acid: a primer on analysis and interpretation. Mar. Mammal Sci. 22, 759–801. 10.1111/j.1748-7692.2006.00079.x

[B4] Crespo B. Teixeira I. Figueiras F. Castro C. (2008). Microplankton composition off NW Iberia at the end of the upwelling season: source areas of harmful dinoflagellate blooms. Mar. Ecol. Prog. Ser. 355, 31–43. 10.3354/meps07261

[B5] Dalsgaard J. St. John M. Kattner G. Müller-Navarra D. Hagen W. (2003). Fatty acid trophic markers in the pelagic marine environment. Adv. Mar. Biol. 46, 225–340. 10.1016/S0065-2881(03)46005-7 14601414

[B6] Estefanell J. Socorro J. Izquierdo M. Roo J. (2015). Effect of two fresh diets and sexual maturation on the proximate and fatty acid profile of several tissues in *Octopus vulgaris* : specific retention of arachidonic acid in the gonads. Aquacult. Nutr. 21, 274–285. 10.1111/anu.12163

[B7] Estefanell J. Socorro J. Ramirez B. Izquierdo M. Roo J. (2013). Fatty acid profile in eggs and newly hatched paralarvae of *Octopus vulgaris* collected from the wild and after 1-5 days starvation. Commun. Agric. Applied Biol. Sci. 78, 119–122. 10.13140/2.1.4164.8644 25141643

[B8] FAO (2016). Fisheries and Aqauculture software. FishStatJ – Software for Fisheries Statistical Time Series, in FAO Fisheries and Aquaculture Department (online). Rome (cited 12 November 2016).

[B9] Falk-Petersen S. Dahl T. Scott C. Sargent J. R. Gulliksen B. Kwasniewski S. (2002). Lipid biomarkers and trophic linkages between ctenophores and copepods in Svalbard waters. Mar. Ecol. Prog. Ser. 227, 187–194. 10.3354/meps,227187

[B10] Fernández-Reiriz M. J. Perez-Camacho A. Ferreiro M. J. Blanco J. Planas M. Campos M. J. (1989). Biomass production and variation in the biochemical profile (Total protein, carbohydrates, RNA, lipids and FA) of seven species of marine microalgae. Aquaculture 83, 17–27. 10.1016/0044-8486(89)90057-4

[B11] Freeman C. T. West D. (1966). Complete separation of lipid classes on a single thin-layer plate. J. Lipid Res. 7, 324–327. 5947043

[B12] Garrido D. Martín V. M. Rodríguez C. Iglesias J. Navarro J. C. Estévez A. (2016a). Meta-analysis approach to the effects of live prey on the growth of *Octopus vulgaris* paralarvae under culture conditions. Rev. Aquacult. 1–12. 10.1111/raq.12142

[B13] Garrido D. Navarro J. C. Perales-Raya C. Nande M. Martín M. V. Iglesias J. (2016b). Fatty acid composition and age estimation of wild *Octopus vulgaris* paralarvae. Aquaculture 464, 564–569. 10.1016/j.aquaculture.2016.07.034

[B14] Garrido S. Rosa R. Ben-Hamadou R. Cunha M. E. Chícharo M. A. van der Lingen C. D. (2008). Spatio-temporal variability in fatty acid trophic biomarkers in stomach contents and muscle of iberian sardine (*Sardina pilchardus*) and its relationship with spawning. Mar. Biol. 154, 1053–1065. 10.1007/s00227-008-0999-7

[B15] Gonçalves A. M. M. Azeiteiro U. M. Pardal M. A. De Troch M. (2012). Fatty acid profiling reveals seasonal and spatial shifts in zooplankton diet in a temperate estuary. Estuar. Coastal Shelf. Sci. 109, 70–80. 10.1016/j.ecss.2012.05.020

[B16] González A. F. Otero J. Guerra Á. Prego R. Rocha F. Dale A. W. (2005). Distribution of common octopus and common squid paralarvae in a wind-driven upwelling area (Ria of Vigo, northwestern Spain). J. Plankton Res. 27, 271–277. 10.1093/plankt/fbi001

[B17] Guerra Á. Hernández-Urcera J. Garci M. E. Sestelo M. Regueira M. González A. F. (2015). Spawning habitat selection by *Octopus vulgaris*: new insights for a more effective management of this resource. Fish. Res. 167, 313–322. 10.1016/j.fishres.2015.03.011

[B18] Iglesias J. Fuentes L. Sánchez J. Otero J. J. Moxica C. Lago M. J. (2006). First feeding of *Octopus vulgaris* cuvier 1797 paralarvae using Artemia: effect of prey size, prey density and feeding frequency. Aquaculture 261, 817–822. 10.1016/j.aquaculture.2006.08.002

[B19] Iglesias J. Pazos G. Fernández J. Sánchez F. J. Otero J. J. Domingues P. (2013). The effects of using crab zoeae (*Maja brachydactyla*) on growth and biochemical composition of *Octopus vulgaris* (Cuvier 1797) paralarvae. Aquacult. Int. 22, 1041–1051. 10.1007/s10499-013-9725-7

[B20] Iglesias J. Sánchez F. J. Bersano J. G. F. Carrasco J. F. Dhont J. Fuentes L. (2007). Rearing of *Octopus vulgaris* paralarvae: present status, bottlenecks and trends. Aquaculture 266, 1–15. 10.1016/j.aquaculture.2007.02.019

[B21] Jackson G. D. Meekan M. G. Wotherspoon S. Jackson C. H. (2008). Distribution of young cephalopods in the tropical waters of western Australia over two consecutive summers. ICES J. Mar. Sci. 65, 140–147. 10.1093/icesjms/fsm186

[B22] Lee R. E. Hagen W. Kattner G. (2006). Lipid storage in marine zooplankton. Mar. Ecol. Prog. Ser. 307, 273–306. 10.3354/meps307273

[B23] Lee R. F. Nevenzel J. C. Paffenhöfer G.-A. (1971). Importance of wax esters and other lipids in the marine food chain: phytoplankton and copepods. Mar. Biol. 9, 99–108. 10.1007/BF00348249

[B24] Lee R. F. Nevenzel J. C. Paffenhöfer G.-A. Benson A. A. (1970). The metabolism of wax esters and other lipids by the marine copepod *Calanus helgolandicus*. J. Lipid Res. 11, 237–240. 5441249

[B25] Lepage G. Roy C. C. (1984). Improved recovery of fatty acid through direct transesterification without prior extraction or purification. J. Lipid Res. 25, 1391–1396. 6530596

[B26] Letessier T. Pond D. McGill R. Reid W. Brierley A. (2012). Trophic interaction of invertebrate zooplankton on either side of the charlie gibbs fracture zone/subpolar front of the mid-atlantic ridge. J. Mar. Syst. 94, 174–184. 10.1016/j.jmarsys.2011.11.020

[B27] Lourenço S. Narciso L. Gonzalez Á. F. Pereira J. Auborg S. Xavier J. C. (2014). Does the trophic habitat influence the biochemical quality of the gonad of *Octopus vulgaris*? Stable isotopes and lipid class contents as bio-indicators of different life-cycle strategies. Hydrobiologia 725, 33–46. 10.1007/s10750-013-1717-0

[B28] Marsh J. B. Weinstein D. B. (1966). Simple charring method for determination of lipids. J. Lipid Res. 7, 574–576. 5965305

[B29] Martínez R. López-Ripoll E. Avila-Poveda O. Santos-Ricalde R. Mascaró M. Rosas C. (2011). Cytological ontogeny of the digestive gland in post-hatching Octopus maya, and cytological background of digestion in juveniles. Aquat. Biol. 11, 249–261. 10.3354/ab00305

[B30] Miliou H. Fintikaki M. Tzitzinakis M. Kountouris T. Verriopoulos G. (2006). Fatty acid composition of the common octopus, *Octopus vulgaris*, in relation to rearing temperature and body weight. Aquaculture 256, 311–322. 10.1016/j.aquaculture.2006.02.050

[B31] Moguel C. Mascaró M. Avila-Poveda O. Caamal-Monsreal C. Sanchez A. Pascual C. (2010). Morphological, physiological and behavioral changes during post-hatching development of Octopus maya (Mollusca: Cephalopoda) with special focus on the digestive system. Aquat. Biol. 9, 35–48. 10.3354/ab00234

[B32] Monroig Ó. Guinot D. Hontoria F. Tocher D. R. Navarro J. C. (2012). Biosynthesis of essential fatty acids in *Octopus vulgaris* (Cuvier, 1797): molecular cloning, functional characterisation and tissue distribution of a fatty acyl elongase. Aquaculture 360–361, 45–53. 10.1016/j.aquaculture.2012.07.016

[B33] Monroig Ó. Tocher D. Navarro J. (2013). Biosynthesis of polyunsaturated fatty acids in marine invertebrates: recent advances in molecular mechanisms. Mar. Drugs 11, 3998–4018. 10.3390/md11103998 24152561 PMC3826146

[B34] Moreno A. dos Santos A. Piatkowski U. Santos A. M. P. Cabral H. (2009). Distribution of cephalopod paralarvae in relation to the regional oceanography of the western Iberia. J. Plankton Res. 31, 73–91. 10.1093/plankt/fbn103

[B35] Moreno A. Lourenço S. Pereira J. Gaspar M. B. Cabral H. N. Pierce G. J. (2014). Essential habitats for pre-recruit *Octopus vulgaris* along the portuguese coast. Fish. Res. 152, 74–85. 10.1016/j.fishres.2013.08.005

[B36] Navarro J. C. Villanueva R. (2000). Lipid and fatty acid composition of early stages of cephalopods: an approach to their lipid requirements. Aquaculture 183, 161–177. 10.1016/S0044-8486(99)00290-2

[B37] Navarro J. C. Villanueva R. (2003). The fatty acid composition of *Octopus vulgaris* paralarvae reared with live and inert food: deviation from their natural fatty acid profile. Aquaculture 219, 613–631. 10.1016/S0044-8486(02)00311-3

[B38] Navarro J. C. Monroig O. Sykes A. V. (2014). Nutrition as a key factor for cephalopod aqua-culture, in Cephalopod Culture, eds Iglesias J. Fuentes L. Villanueva R. (New York, NY: Springer), 77–96.

[B39] Oksanen J. Blanchet F. G. Kindt R. Legendre P. Minchin P. R. O'hara R. B. (2013). Vegan: Community Ecology Package. R Package Version 2.0-10. Available online at: http://CRAN.R-project.org/package=vegan

[B40] Okumura S. Kurihara A. Iwamoto A. Takeuchi T. (2005). Improved survival and growth in *Octopus vulgaris* paralarvae by feeding large type Artemia and Pacific sandeel, Ammodytes personatus. Aquaculture 244, 147–157. 10.1016/j.aquaculture.2004.11.044

[B41] Olmos-Pérez L. Roura Á. Pierce G. J. Boyer S. González Á. F. (2017). Diet composition and variability of wild *Octopus vulgaris* and Alloteuthis media (Cephalopoda) paralarvae through a metagenomic lens. Front. Physiol. 8:321. 10.3389/fphys.2017.00321 28596735 PMC5442249

[B42] Otero J. Álvarez-Salgado X. González Á. F. Gilcoto M. Guerra Á. (2009). High-frequency coastal upwelling events influence *Octopus vulgaris* larval dynamics on the NW Iberian shelf. Mar. Ecol. Prog. Ser. 386, 123–132. 10.3354/meps08041

[B43] Otero J. Álvarez-Salgado X. González Á. F. Miranda A. Groom S. B. Cabanas J. M. (2008). Bottom-up control of common Octopus *Octopus vulgaris* in the Galician upwelling system, northeast Atlantic Ocean. Mar. Ecol. Prog. Ser. 362, 181–192. 10.3354/meps07437

[B44] Passarella K. C. Hopkins T. L. (1991). Species composition and food habits of the micronektonic cephalopod assemblage in the Eastern Gulf of Mexico. B. Mar. Sci. 49, 638–659.

[B45] Quintana D. Márquez L. Arévalo J. R. Lorenzo A. Almansa E. (2015). Relationships between spawn quality and biochemical composition of eggs and hatchlings of *Octopus vulgaris* under different parental diets. Aquaculture 446, 206–216. 10.1016/j.aquaculture.2015.04.023

[B46] Reis D. B. Acosta N. G. Almansa E. Navarro J. C. Tocher D. R. Monroig O. (2014). *In vivo* metabolism of unsaturated fatty acids in *Octopus vulgaris* hatchlings determined by incubation with 14C-labelled fatty acids added directly to seawater as protein complexes. Aquaculture 431, 28–33. 10.1016/j.aquaculture.2014.05.016

[B47] Reis D. B. García-Herrero I. Riera R. Felipe B. C. Rodríguez C. Sykes A. V. (2015). An insight on *Octopus vulgaris* paralarvae lipid requirements under rearing conditions. Aquacult. Nutr. 21, 797–806. 10.1111/anu.12205

[B48] Rosa R. Costa P. R. Nunes M. L. (2004). Effect of sexual maturation on the tissue biochemical composition of *Octopus vulgaris* and *O. defilippi* (Mollusca: Cephalopoda). Mar. Biol. 145, 563–574. 10.1007/s00227-004-1340-8

[B49] Roura Á. Álvarez-Salgado X. A. González Á. F. Gregori M. Rosón G. Guerra Á. (2013). Short-term meso-scale variability of mesozooplankton communities in a coastal upwelling system (NW Spain). Prog. Oceanogr. 109, 18–32. 10.1016/j.pocean.2012.09.003

[B50] Roura Á. Álvarez-Salgado X. A. González Á. F. Gregori M. Rosón G. Otero J. (2016). Life strategies of cephalopod paralarvae in a coastal upwelling system (NW Iberian Peninsula): insights from zooplankton community and spatio-temporal analyses. Fish. Oceanogr. 25, 241–258. 10.1111/fog.12151

[B51] Roura Á. González Á. F. Pascual S. Guerra Á. (2010). A molecular approach to identifying the prey of cephalopod paralarvae. ICES J. Mar. Sci. 67, 1408–1412. 10.1093/icesjms/fsq051

[B52] Roura Á. González Á. F. Redd K. Guerra Á. (2012). Molecular prey identification in wild *Octopus vulgaris* paralarvae. Mar. Biol. 159, 1335–1345. 10.1007/s00227-012-1914-9

[B53] Seixas P. Rey-Méndez M. Valente L. M. Otero A. (2008). Producing juvenile Artemia as prey for *Octopus vulgaris* paralarvae with different microalgal species of controlled biochemical composition. Aquaculture 283, 83-91. 10.1016/j.aquaculture.2008.06.019

[B54] Seixas P. Rey-Méndez M. Valente L. M. P. Otero A. (2010). High DHA content in Artemia is ineffective to improve *Octopus vulgaris* paralarvae rearing. Aquaculture 300, 156–162. 10.1016/j.aquaculture.2009.12.021

[B55] Sieiro M. P. Aubourg S. P. Rocha F. (2006). Seasonal study of the lipid composition in different tissues of the common octopus (*Octopus vulgaris*). Eur. J. Lipid Sci. Technol. 108, 479–487. 10.1002/ejlt.200500322

[B56] Tocher D. R. Glencross B. D. (2015). Lipids and fatty acids, in Dietary Nutrients, Additives, and Fish health, eds Lee C.-S. Lim C. Gatlin D. M. Webster C. D. (Hoboken, NJ: Wiley Blackwell), 47–94.

[B57] Vaz-Pires P. Seixas P. Barbosa A. (2004). Aquaculture potential of the common octopus (*Octopus vulgaris* Cuvier, 1797): a review. Aquaculture 238, 221–238. 10.1016/j.aquaculture.2004.05.018

[B58] Viciano E. Iglesias J. Lago M. J. Sánchez F. Otero J. Navarro J. C. (2011). Fatty acid composition of polar and neutral lipid fractions of *Octopus vulgaris* Cuvier, 1797 paralarvae reared with enriched on-grown Artemia: polar and neutral lipid fatty acids of octopus. Aquac. Res. 42, 704–709. 10.1111/j.1365-2109.2010.02605.x

[B59] Villanueva R. (1994). Decapod crab zoeae as food for rearing cephalopod paralarvae. Aquaculture 128, 143–152. 10.1016/0044-8486(94)90109-0

[B60] Villanueva R. Nozais C. Boletzky S. V. (1996). Swimming behaviour and food searching in planktonic *Octopus vulgaris* Cuvier from hatching to settlement. J. Exp. Mar. Biol. Ecol. 208, 169–184. 10.1016/S0022-0981(96)02670-6

[B61] Zaragoza N. Quetglas A. Moreno A. (2015). Identification guide for Cephalopod Paralarvae from the Mediterranean Sea. ICES Cooperative Research Report.

[B62] Zuur A. F. Ieno E. N. Smith G. M. (2007). Analysing Ecological Data. New York, NY: Springer.

